# The impact of carbon information disclosure on corporate green technology innovation in the context of “dual carbon”—— Based on data from heavily polluting industries

**DOI:** 10.1371/journal.pone.0319997

**Published:** 2025-03-13

**Authors:** Lumenqiqige Chao, Richa Hu, Boya Shi, Xiaomin Jin

**Affiliations:** 1 School of Accountancy, Inner Mongolia University of Finance and Economics, Hohhot, China; 2 School of Business Administration, Zhongnan University of Economics and Law, Wuhan, China; Brunei University London, UNITED KINGDOM OF GREAT BRITAIN AND NORTHERN IRELAND

## Abstract

In the current global response to climate change, the “dual carbon” goal has become a common development goal for all countries. In the context of “dual carbon”, China’s heavily polluting enterprises, as the main governance body, have become the necessary path for green transformation. Green technology innovation is important for heavily polluting industries to achieve green transformation. Starting from the perspective of environmental regulation based on information disclosure, this article mainly explores the mechanism of carbon information disclosure promoting green technology innovation in enterprises. This article takes heavily polluting enterprises in China from 2017 to 2021 as samples and innovatively constructs a carbon information disclosure indicator system based on the “dual carbon” background. Then, empirical tests examine the relationship between carbon information disclosure level, green technology innovation, and financing constraints. Research has shown that the quality of carbon information disclosure improves corporate green technology innovation; The quality of carbon information disclosure can promote green technology innovation by alleviating corporate financing constraints. The research results have theoretical value and significance for incentivizing heavily polluting enterprises to reduce carbon emissions and support China’s dual carbon goals.

## Introduction

In the context of global warming, energy conservation and emission reduction, low-carbon economy, and green development have become a global consensus. Governments of various countries actively participate in global climate governance and have achieved important results such as the Kyoto Protocol and the Paris Agreement. In the current global response to climate change, the “dual carbon” goal has become a common development goal for all countries, which is to achieve carbon peak and carbon neutrality by the middle of this century [1]. In 2021, at the National People’s Congress and Chinese People’s Political Consultative Conference [2], China proposed for the first time the goal of achieving carbon peak and carbon neutrality and further included it in the 14th Five-Year Plan. The Chinese government has also been working hard to control greenhouse gas emissions [3]. In December 2017, China embarked on establishing a pilot national carbon emissions trading market, which was officially launched in 2022. The proposition of the “dual carbon” target is not only a testament to the evolution of our times but also represents a crucial pathway for China’s transition towards a green economy [4]. The report of the 20th National Congress of the Communist Party of China emphasizes the importance of accelerating the green transformation of the development mode, intensifying efforts in environmental pollution prevention and control, and actively yet steadily pursuing carbon peaking and carbon neutrality [5]. To achieve this, it proposes enhancing the regulation of total energy consumption and intensity, with a particular focus on controlling fossil energy consumption. Furthermore, it advocates a gradual transition towards a dual control system that encompasses both carbon emissions and intensity, marking a significant stride towards a more sustainable and environmentally friendly future [6].

Heavy polluting enterprises, as the main body of governance, are the main source of energy consumption and greenhouse gas emissions of carbon dioxide [7]. Green technology innovation is an important means for heavily polluting industries to achieve green transformation. On the one hand, green technology innovation is the main driving force for promoting green development. Realizing the carbon peak goal inevitably means adjusting the high carbon energy structure, upgrading industries, comprehensively conserving energy, and reducing emissions [8]. On the other hand, targeting high-polluting enterprises and carrying out green technology innovation can not only generate significant economic benefits but also significant environmental benefits, which is of great practical significance for the country to achieve “low-carbon” development [9]. Unlike traditional innovation, green technology innovation pursues a win-win situation that can generate economic benefits while protecting the environment. As an important contributor to carbon reduction, heavily polluting enterprises face higher requirements for their development [10]. They need to focus on increasing research and development efforts, improving core technology research capabilities, carrying out green technology innovation, and achieving green transformation to support the dual carbon cause [11]. However, compared with foreign countries, China has yet to establish a unified standard for carbon information disclosure. Consequently, enterprises in China exhibit a relatively weak willingness and initiative in disclosing carbon information. This lack of standardization and enthusiasm poses challenges in monitoring and managing carbon emissions effectively, hindering progress towards achieving carbon peaking and carbon neutrality goals [12]. Therefore, many enterprises have varying degrees of problems in carbon accounting information disclosure, and some listed companies have not even disclosed relevant information [13,14], further exacerbating the information asymmetry between enterprises and external stakeholders [15,16]. Reasonable and effective disclosure of carbon information can help companies recognize their strengths and weaknesses in carbon management [17], cultivate awareness of coordinated development between energy conservation and emission reduction and economic benefits, provide investors with information on corporate carbon emissions and carbon assets [18], and help investors have a deeper understanding and supervision of corporate business behavior [19], thus providing stable financial support for enterprises, and ultimately affecting enterprises to carry out technological innovation activities [20,21]. Therefore, our study examines the impact of carbon information disclosure quality of heavily polluting enterprises on their green technology innovation and analyzes its mechanism using financing constraints as a mediating variable. Listed companies within the heavily polluting industries of the Shanghai and Shenzhen stock markets were chosen as the subjects of the research [22], and empirically test the impact of corporate carbon disclosure information quality on green technology innovation, and further verify the mediating effect of financing constraints.

The possible contributions of the article are as follows: First, this study significantly contributes to the literature on green technology innovation. Based on environmental information disclosure, a new type of environmental regulation, this paper analyzes the internal mechanism of carbon information disclosure affecting enterprise green technology innovation from the perspective of financing constraints, so as to make a positive discussion in theory. Second, the construction of carbon information disclosure quality indicators based on the China dual carbon target strategy is established, which enriches the content of environmental information disclosure by enterprises and provides ideas for accurate, reliable, and comprehensive information disclosure systems, to empirically analyze the influence effect and direction of carbon information disclosure on green technology innovation of enterprises. Third, this study focuses on Chinese heavily polluting enterprises, which are high carbon emission sources in China. The research results have important reference significance and value for achieving the dual carbon goals at the level of Chinese heavily polluting enterprises.

## Literature review

### Research on the quality of carbon information disclosure and green technology innovation

Carbon information disclosure belongs to a policy that achieves environmental regulation through information disclosure. Therefore, this study reviews green innovation from the perspective of environmental disclosure [23]. Carbon information disclosure is also known as carbon accounting information disclosure. Usually, it refers to the behavior of greenhouse gas emitting units to truthfully, comprehensively, timely, and fully disclose carbon emission-related information to investors and the public in the form of regular reports and temporary reports [24]. Scholars explore the impact of different information disclosure policies on corporate technological innovation [25].

Relevant studies have shown that China’s carbon emission trading pilot policies have promoted green innovation among enterprises in pilot areas [26,27]. Other studies have shown that the consistency between corporate carbon performance and carbon information disclosure will have a more significant positive impact on corporate value [28]. The likelihood of large enterprises promoting green technology innovation and green operation innovation through disclosure of carbon emission information to improve carbon performance is significantly higher than that of small and medium-sized enterprises [29]. Companies that excel in both environmental governance and digital technology tend to pursue green innovation to address climate risks [30]. Study find that good corporate social responsibility performance has a significant positive impact on the number of green patent applications and authorizations of enterprises [31]. For heavily polluting enterprises in our country, providing environmental information, including carbon information disclosure, can reduce information asymmetry between stakeholders and enterprise managers, improve the reputation of enterprises in the environment, alleviate financing constraints [32], and better encourage enterprises to engage in technological innovation related to energy conservation and environmental protection [33]. This will help improve energy efficiency, reduce carbon emissions, and thus enhance the level of green technology innovation in enterprises. At present, there is a lack of research on the quality of carbon information disclosure in existing studies that combines environmental information disclosure with the “dual carbon” goals, as well as related research focusing on green technology innovation in enterprises, which still needs to be continuously enriched and deepened [34].

### Research on the intermediary role of financing constraints

Enterprises need to invest a large amount of funds in technology research and development when carrying out green technology innovation, but its risks are high, the cycle is long, and the degree of information asymmetry is high. Enterprises need to have strong capital investment capabilities while also facing challenges brought about by market and product uncertainty, making it difficult to achieve satisfactory economic benefits in the short term and thus easily facing serious financing constraints [20]. This financing constraint is caused by market incompleteness, such as information asymmetry and agency problems. Scholars have shown that there are indeed certain financing constraints on green technology innovation activities of enterprises, which limit their development [35]. However, some scholars have also suggested that companies actively disclosing carbon information will significantly alleviate their external financing constraints [36]. It is believed that improving the quality of corporate social responsibility information disclosure can accelerate the flow and sharing of information, thereby better connecting the supply and demand sides of funds, and reducing the financing constraints faced by companies in technological innovation activities [37]. The disclosure of environmental performance and practices by enterprises to external investors can generate an information increment effect, enabling investors to better predict the future business development of the enterprise and reduce information collection and transaction costs, thereby alleviating the financing constraints faced by the enterprise.

## Theoretical analysis and research hypotheses

### The quality of carbon information disclosure and corporate green technology innovation

In the context of dual carbon, environmental regulation is becoming increasingly strict, and the impact of green innovation on corporate competition is becoming increasingly important. Enterprise green innovation refers to the collective activity of enterprises to innovate products or production processes for energy conservation and emission reduction while improving the utilization rate of production materials. The green technology innovation of enterprises is similar to general technology innovation, with characteristics such as uncertain profits and strong confidentiality. Especially for heavily polluting enterprises, green technology innovation is an important means of their green transformation and development. However, green technology innovation in enterprises is often constrained by funding shortages, high failure risks, and insufficient innovation motivation.

Based on the theory of information asymmetry, the information barriers between enterprises and external investors make it difficult for investors to accurately understand the efforts made by enterprises for green technology innovation, thereby increasing the risk of adverse selection [38]. Carbon information disclosure can expand the information scope of enterprise production and operation as well as low-carbon emission reduction, help external investors understand the development of enterprise green technology innovation activities and make reasonable decisions, and provide financial support for enterprise green technology innovation. Carbon information disclosure, as an important component of social responsibility, can reflect the low-carbon emission reduction responsibility undertaken by enterprises, convey positive information about green operations to the outside world, and establish a corporate image with a high sense of social responsibility. While attracting more social capital, it can also attract more innovative talents and promote green technology innovation in enterprises.

Related studies have shown that under strict environmental regulations and supervision, negative environmental pollution incidents of heavily polluting listed companies will further deteriorate their reputation, leading to a chain reaction of environmental department regulation and stock market sell-off [39]. Therefore, more and more enterprises are inclined to improve the quality of carbon information disclosure to cope with the legitimacy of the government and the public, as well as the pressure of environmental energy conservation and low-carbon expectations, which is conducive to green technology innovation. Meanwhile, the high-quality carbon information disclosed by enterprises will attract more and more environmental investors [40]. Their focus on low-carbon business practices and demand for environmentally friendly products will in turn encourage companies to optimize production processes and innovate green technologies. It can be seen that carbon information disclosure can promote green technology innovation in enterprises by alleviating information asymmetry, improving corporate reputation, and strengthening legitimacy. On this basis, this article proposes the following assumptions:

Hypothesis 1: The quality of corporate carbon information disclosure can effectively promote green technology innovation.

### The intermediary role of financing constraints

The technological innovation capability of enterprises mainly depends on their R&D investment to a certain extent, and the social benefits of green technology innovation in enterprises are greater than private benefits, which can easily lead to insufficient capital investment [41]. Existing research suggests that financing constraints, government subsidies, and other factors may be possible ways in which the quality of carbon information disclosure affects corporate green technology innovation [42]. Enterprises usually proactively disclose environmental and carbon accounting information to reduce information asymmetry with stakeholders and promote their green development, especially when external financial support is needed. Considering the current situation of financing constraints faced by heavily polluting enterprises in China, this article explores the impact of carbon information disclosure quality on green technology innovation of enterprises from the perspective of financing constraints.

Based on stakeholder theory, corporate investors and debtors are more inclined to invest in companies with good reputations and promising future development prospects. The carbon accounting information disclosed by the company can meet the information needs of stakeholders for decision-making. Enterprise innovation activities cannot be separated from long-term stable financial support. The source of enterprise funds comes from both internal funds for business operations and external funds such as financial markets and banking institutions. Under the dual carbon target strategy, heavily polluting enterprises need to carry out green production and operation, guide funds to invest in green development enterprises, and thus affect the financing and investment decisions of heavily polluting enterprises [43].

In addition, due to information barriers between enterprises and investors, investors often attach restrictive clauses, which increases the financing costs of enterprises. In the current financial market of our country, there are three main channels for the generation and dissemination of information: information disclosed by listed companies, information provided by financial intermediaries, and the general market. Due to the imperfect financial market and insufficient information transmission in our country [44]. Therefore, improving the level of enterprise information disclosure can effectively alleviate the problem of information asymmetry. Scholars found that information disclosure can significantly improve the transparency of enterprise information, reduce financing costs, improve business performance, and promote innovation [45,46]. Similarly, Wang et al. [47] have empirically verified that different types of social responsibility information disclosure can alleviate corporate financing constraints, help obtain external funds to alleviate financing constraints and promote technological innovation. On this basis, this article proposes the following assumptions:

Hypothesis 2: The quality of corporate carbon information disclosure can promote green technology innovation by alleviating financing constraints.

**Fig 1:**
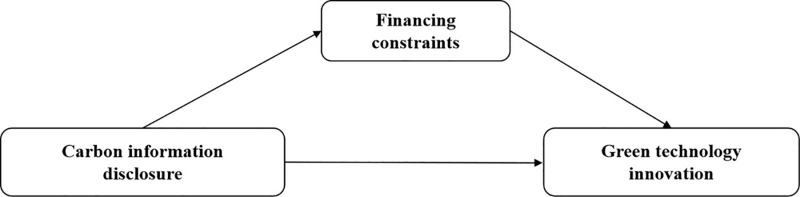
Theoretical model.

To sum up, the theoretical model constructed in this paper is shown in Fig 1.

### Research design

#### Data sources.

In this study, we select the relevant data of all A-share heavy polluting industries in the Shanghai and Shenzhen stock markets from 2017 to 2021 as the sample. According to the “Guidelines for Environmental Information Disclosure of Listed Companies” issued by the Ministry of Environmental Protection, China’s current heavy polluting industries include a total of sixteen categories, including thermal power, steel, cement, electrolytic aluminum, coal, metallurgy, etc. Excluding companies with ST and *  ST during this period, companies that did not disclose green innovation-related data, and companies with incomplete financial indicators, 2324 valid observation samples were obtained. The carbon information disclosure data mainly comes from the environmental research database in the China Stock Market & Accounting Research Database (CSMAR), and the green technology innovation data comes from the green patent database in the China Research Data Service Platform (CNRDS). Other indicators such as asset liability ratio and profitability were taken from the CSMAR database. To eliminate the influence of extreme values, all variables were truncated.

### Variable definition

**Explained variable.** Green technology innovation. We use the natural logarithm of patent authorization and repeated logarithms to measure green technology innovation, including green inventions and green utility patents. Although patent application data updates quickly, the number of green patent authorizations can better represent the true innovation level of enterprises [48].

**Explanatory variables.** Quality of carbon information disclosure. We have constructed a quality evaluation system for corporate carbon information disclosure based on previous research results [49,50]. When determining specific evaluation assignment dimensions and consideration indicators: First, refer to the settings of major authoritative international organizations for corporate carbon information disclosure management, including the CDP (Carbon Information Disclosure) project, the Climate Change Reporting Framework issued by the Climate Disclosure Standards Board (CDSB), and the Global Climate Risk Disclosure Framework issued by the Climate Risk Disclosure Initiative (CRDI), and draw on their requirements for corporate carbon information management and reporting; Secondly, referring to the “General Principles for Accounting and Reporting of Greenhouse Gas Emissions from Industrial Enterprises” released in 2015 in China, it is stipulated that enterprise reports should include basic information on the subject, accounting, and the total amount of greenhouse gas emissions during the reporting period. Therefore, this article explores the entire process of enterprise carbon reduction management activities, from the overall strategic goals to the action performance and organizational management and incentives at the execution level, and finally to the carbon emission accounting and trading at the results level, and determines an indicator system consisting of 4 primary indicators and 12 secondary indicators, as shown in Table 1. This article draws on the approach of scholars [51,52], uses content analysis to obtain indicator values, and scores each evaluation indicator. The established indicator system aims to evaluate corporate carbon information disclosure from the perspective of information quality. Therefore, drawing on the ideas of accounting information quality characteristics mentioned in China’s “Enterprise Accounting Standards - Basic Standards”, four dimensions of significance, timeliness, reliability, and quantification are selected for scoring [53]. The final score of this indicator system is between 0-16 points, and the specific assignment basis is shown in Table 2. The specific formula for scoring the quality of corporate carbon information disclosure is as follows:

**Table 1:** Quality Evaluation Index System for Carbon Information Disclosure.

Primary Indicators	Secondary Indicators	Indicator Connotation
Carbon emission management disclosure	Environmental protection concept	The emission reduction planning content, carbon emission target details, plans formulated to achieve low-carbon development, procurement of carbon reduction equipment, low-carbon technology research and development, commercial cooperation, and training included in the enterprise strategy.
	Environmental goals	
	Environmental management system	
	Environmental education and training	
Carbon emission regulation and certification disclosure	Has it passed ISO14002 certification?	Heavy polluting enterprises obtain various certifications and pollutant standards to achieve the expected goals of low-carbon action.
	Has it passed ISO9001 certification?	
	Pollutant discharge meets standards	
Environmental information disclosure	Annual report of listed companies	Complete disclosure elements and proactive disclosure of various financial statements, representing a certain degree of corporate carbon reduction responsibility and action.
	Social responsibility report	
	Environmental report	
Carbon emission performance and governance disclosure	Situation of waste gas emission reduction and control	Measure the degree of low-carbon progress of enterprises through specific implementation actions, disclose the actual low-carbon operations and low-carbon governance investment of enterprises.
	Implementation of clean production	

**Table 2:** Detailed Rules for Quality Scoring of Carbon Information Disclosure.

Evaluation Dimension	Judgment Basis	Scoring Rubric
Significance	Types of reports on the quality of carbon information disclosure	Disclosure earns 1 point, otherwise 0 points.
Timeliness	The time dimension of carbon information reflection	When the information is not disclosed, it is recorded as “0” points, and when a qualitative description of the item is made, it is recorded as “1” points.
Reliability	Do you have an independent third-party inspection certificate	When the information is not disclosed, it is recorded as “0” points, and when a qualitative description of the item is made, it is recorded as “1” points.
Quantitative Ness	Qualitative or quantitative description, whether it involves monetary information	When the item is not disclosed, it is recorded as 0 points. When a qualitative description of the information is given, it is recorded as 1 point. When a quantitative description of the indicator is given in monetary or numerical terms, it is recorded as 2 points.

Quality of corporate carbon information = Score for corporate carbon information disclosure/ Total score for carbon information disclosure

**Mediating variable.** Financing constraints. This article selects the SA index as an indicator to measure corporate financing constraints [54], with the specific formula shown in Table 3, and takes the absolute value of the SA index.

**Table 3:** Variable Definition and Interpretation.

Variable Type	Variable Name	Variable Symbol	Variable Definition
**Explained Variable**	Green technology innovation	GTI	The number of green patent applications obtained from the green patent database of Chinese listed companies is logarithmic to the sum of 1.
**Explanatory variable**	Quality of carbon information disclosure	QCID	Establish a carbon information evaluation index system to determine the quality of carbon information disclosure.
**Mediating variable**	Financing constraints	SA	SA:Size = -0.737*Size + 0.043 * Size2-0.04 * Age
**Control variable**	Profit ability	ROA	Net profit at the end of the period divided by total assets at the end of the period.
	Cash flow from operations	CASH	Cash flow from operating activities divided by total assets at the end of the period.
	Asset liability ratio	LEV	Total liabilities at the end of the period divided by total assets.
	Years of establishment of the enterprise	AGE	The logarithm of the establishment time of the enterprise.
	Board size	BOARD	Number of directors in pairs.

**Control variables.** This article draws on previous scholars’ research and focuses on other main factors that affect the level of green innovation in enterprises [55]. It selects asset-liability ratio (LEV), profitability (ROA), cash flow from operating activities (CASH), board size (BOARD), and age of establishment (AGE) as control variables for research [56].

### Model construction

To empirically test whether the quality of carbon information disclosure promotes green technology innovation in enterprises, the following model is constructed:


$$GTI_{it}=\alpha_{0}+\alpha_{1}QCID+\alpha_{2}Controls_{it}+\text{Year}+Industr\text{y}+\varepsilon_{it}$$ (1)

In model (1), if the regression coefficient before QCID is significantly positive, it indicates that the quality of carbon information disclosure has a significant promoting effect on the level of green technology innovation of enterprises, and verifies hypothesis 1 of this study.

To further examine whether financing constraints affect the promoting effect of carbon information disclosure quality on green technology innovation in enterprises, this paper uses the three-step mediation effect method proposed by previous research to establish a mediation effect model and constructs the following research model:


$$SA_{it}=\beta_{0}+\beta_{1}QCID_{it}+\beta_{2}Controls_{it}+\text{Year}+Industr\text{y}+\varepsilon_{it}$$ (2)

$$GTI_{it}=\gamma_{0}+\gamma_{1}QCID_{it}+\gamma_{2}SA+\gamma_{3}Controls_{it}+\text{Year}+Industr\text{y}+\varepsilon_{it}$$ (3)

Among them, Controls represent other control variables that affect green technology innovation in enterprises, ε the random error term.

**Fig 2:**
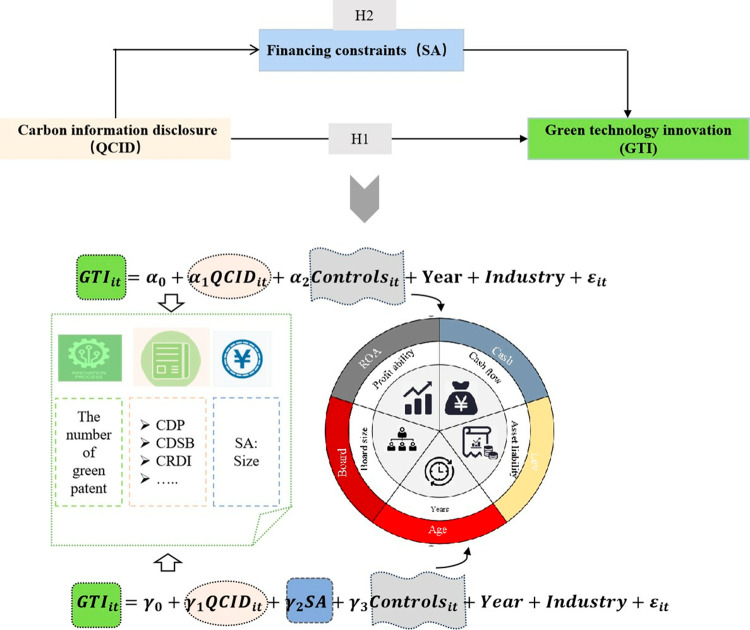
The process of model construction.

To sum up, the process of model construction of this paper is shown in Fig 2.

### Empirical results and analysis

#### Descriptive statistics.

Table 4 presents the descriptive statistical results of each relevant variable. The mean value of Green Technology Innovation (GTI) is 1.402, with a standard deviation of 1.142, a maximum value of 4.394, and a minimum value of 0. This indicates that there is a significant gap in green technology innovation among the sample enterprises, and overall, high-polluting enterprises in China have low enthusiasm for applying for green-related technologies. The average quality of carbon information disclosure (QCID) is 0.373, with a maximum value of 0.813 and a minimum value of 0.125. Enterprises that have disclosed carbon information do not have high disclosure quality, and their awareness of carbon information disclosure is still weak. Looking at financing constraints (SA), the average is 3.883, the maximum is 4.395, and the minimum is 2.812. From this, it can be seen that each sample company is facing serious financial constraints, and the gap between them is also relatively large.

**Table 4:** Descriptive Statistics.

Variable	Observations	Mean Value	Median	Standard Deviation	Minimum Value	Maximum Value
GTI	2324	1.402	1.386	1.142	0	4.394
QCID	2324	0.373	0.375	0.168	0.125	0.813
SA	2324	3.883	3.894	0.239	2.812	4.395
ROA	2324	0.050	0.043	0.054	-0.156	0.212
CASH	2324	0.070	0.067	0.061	-0.091	0.250
LEV	2324	0.441	0.446	0.178	0.074	0.848
BOARD	2324	2.157	2.197	0.201	1.609	2.708
AGE	2324	2.997	3.045	0.251	2.303	3.497

### Empirical result analysis

#### Baseline regression.

Table 5 shows the regression results between the quality of corporate carbon information disclosure and the level of green technology innovation. Column (1) shows that the quality of carbon information disclosure can have a significant impact on the level of green innovation of enterprises before excluding various control variables. Column (2) includes various control variables, and it can be seen that the quality of carbon information disclosure has a significant promoting effect on the level of green innovation. The previous coefficient of QCID is positive, which is significant at the 1% level. The quality of carbon information disclosure has a significant promoting effect on the level of green technology innovation of enterprises, and hypothesis 1 has been verified. In addition, there is a significant positive correlation between corporate profitability and green technology innovation capability at the 1% level. Companies with stronger operational capabilities have a higher pursuit of green innovation. Good business management ability is a prerequisite for the sustainable development of enterprises and a necessary prerequisite for achieving green technology innovation.

**Table 5:** Baseline Regression.

Variable	-1	-2
**GTI**	**GTI**
QCID	1.102***	0.865***
	-7.93	-6.22
ROA		2.063***
		-4.15
CASH		0.184
		-0.45
LEV		1.641***
		-11.58
BOARD		0.478***
		-4.19
AGE		-0.198**
		(-2.25)
Constant	0.991***	-0.01
	-17.4	(-0.03)
Observations	2,324	2,324
R-squared	0.026	0.172
Industry FE	NO	YES
Year FE	NO	YES

***, **, and *denote statistical significance at the 1%, 5%, and 10% levels, respectively.

#### Mediation analysis.

This study examines the intermediary effect from the perspective of financing constraints and Columns (1)-(3) present empirical results with financing constraints as the mediating variable. Based on Table 6, Column (1) represents Hypothesis 1, where the regression coefficient $\alpha_{1}$ for carbon information disclosure quality (QCID) is 0.865 and significant at the 1% level. This indicates that companies with higher carbon information disclosure quality have higher levels of green technology innovation, effectively reducing internal and external information asymmetry and enabling companies to better respond to legitimacy pressure from various stakeholders, thus promoting green technology innovation. In Column (2), the regression coefficient $\beta_{1}$ for corporate carbon information disclosure quality (QCID) is 0.190 and significant at the 1% level, demonstrating that corporate carbon information disclosure can help reduce financing constraints. In Column (3), the coefficient $\gamma_{1}$ for corporate carbon information disclosure quality (QCID) is 0.350, and the coefficient $\gamma_{2}$ for financing constraints (SA) is -2.713, both significant at the 1% level.

**Table 6:** Regression Results of Mediation Effect.

Variable	(1)	(2)	(3)
	GTI	SA	GTI
QCID	0.865***	-0.190***	0.350***
	(6.22)	(-10.08)	(2.60)
SA			-2.713***
			(-18.30)
ROA	2.063***	-0.159***	1.633***
	(4.15)	(-3.01)	(3.39)
CASH	0.184	-0.146***	-0.212
	(0.45)	(-3.32)	(-0.54)
LEV	1.641***	-0.197***	1.105***
	(11.58)	(-10.52)	(7.99)
BOARD	0.478***	-0.094***	0.224**
	(4.19)	(-6.28)	(2.12)
AGE	-0.198**	0.766***	1.879***
	(-2.25)	(63.55)	(12.81)
Constant	-0.010	1.837***	4.975***
	(-0.03)	(38.96)	(12.08)
Observations	2,324	2,324	2,324
R-squared	0.172	0.695	0.270
Industry FE	YES	YES	YES
Year FE	YES	YES	YES

***, **, and *denote statistical significance at the 1%, 5%, and 10% levels, respectively.

We will further investigate whether there is a mediating effect and use Bootstrap to test it. According to Table 7, the bootstrap test results show that 0 is not included in the 95% confidence interval, thus rejecting the null hypothesis and exhibiting a mediating effect. According to the calculation of direct and mediating effects, the proportion of mediating effects to the total effect is 59.59%. Consistent with the main regression results of the mediating effect. Referring to columns (1) - (3), it can be seen that the coefficients $\alpha_{1}$ and $\beta_{1}\gamma_{2}$ the two symbols are both positive, indicating that financing constraints play a partial mediating role in the process of carbon information disclosure improving green innovation. According to the theoretical analysis in the previous text, the quality of carbon information disclosure increases the trust of corporate creditors and shareholders by improving corporate information transparency, reduces corporate financing costs, and thus alleviates corporate financing constraints, giving companies the motivation to apply funds to green research and development investment, and promoting corporate green technology innovation. And the regression results support hypothesis 2.

**Table 7:** Bootstrap Inspection Results Table.

Effect	Regression Coefficient	95% Confidence Interval
**Mediation Effect**	0.515***	(0.39, 0.64)
	(0.0625)	
**Direct Effect**	0.350***	(0.09, 0.62)
	(0.133)	

***, **, and *denote statistical significance at the 1%, 5%, and 10% levels, respectively.

### Robustness test

This article will conduct robustness tests using the instrumental variable method and replacing the dependent variable. First, there may be a causal relationship between the quality of corporate carbon information disclosure and green technology innovation, leading to endogeneity issues. Therefore, referring to the selection method of instrumental variables in relevant literature [47], the lagged period of the explanatory variable is used as the instrumental variable. Column (1) in Table 8 shows the first-stage report results using instrumental variables, which is significant at the 1% level with an estimated coefficient of 0.7510, and the F-statistic value is 207.71, much greater than 10, therefore rejecting the null hypothesis of “weak instrumental variable”. It can be considered that the instrumental variable is reasonable. Column (2) shows the second stage results, indicating that corporate carbon information disclosure quality can indeed significantly promote green technology innovation. Second, the number of green patent authorizations was selected and added to 1 before taking the logarithm to measure the level of green technology innovation in enterprises. The results are shown in column (3) of Table 8. The empirical regression results are consistent with the previous results. The quality of carbon information disclosure in enterprises still significantly promotes green technology innovation, and the results are robust.

**Table 8:** Robust Test.

Variable	(1)	(2)	(3)
	Instrumental variable method first phase	Instrumental variable method phase 2	Replace dependent variable
QCID		0.988***	0.923***
		(0.202)	(7.51)
IV	0.7510***		
	(0.015)		
ROA	0.0702	1.901***	0.700
	(0.058)	(0.571)	(1.59)
CASH	0.0706	0.552	0.824**
	(0.048)	(0.473)	(2.35)
LEV	0.0256	1.789***	1.489***
	(0.016)	(0.158)	(11.94)
BOARD	0.0194	0.474***	0.449***
	(0.013)	(0.126)	(4.46)
AGE	-0.0040	-0.087	-0.249***
	(0.011)	(0.105)	(-3.27)
Constant	0.0917**	-1.059***	-0.052
	(0.040)	(0.398)	(-0.18)
Observations	1,857	1,857	2,322
R-squared	0.594	0.142	0.211
Industry FE	YES	YES	YES
Year FE	YES	YES	YES

***, **, and *denote statistical significance at the 1%, 5%, and 10% levels, respectively.

## Conclusion

This study focuses on the impact of corporate carbon information disclosure on green technology innovation and conducts an empirical test using data from China’s Shanghai and Shenzhen A-shares from 2017 to 2021. The findings indicate a promoting effect of carbon information disclosure on green technology innovation. Firstly, this research demonstrates that high-quality carbon information disclosure is positively related to the green technology innovation level of enterprises. This result shows that carbon information disclosure positively influences corporate green technology innovation. For heavily polluting enterprises, high-quality carbon information disclosure can alleviate financing constraints, and thus contribute to the long-term development of the enterprise[57,58]. Secondly, the research finds that alleviating financing constraints plays a partial intermediary role in the relationship between corporate carbon information disclosure and green technology innovation. This result shows that corporate carbon information disclosure promotes green technology innovation by alleviating financing constraints. Our findings have critical theoretical and practical implications. Improving carbon information disclosure quality can alleviate financing constraints and comprehensively promote corporate green technology innovation.

## Research implications

### Theoretical implications

First, this research provides new insights into the drivers of corporate green technology innovation. With the increasing support for technological innovation in China, green technology innovation has become a necessary path for heavily polluting enterprises. Heavy polluting enterprises, as high carbon emitting and highly polluting entities, are a major obstacle to achieving the dual carbon goals. However, existing studies have focused more on the combination of green innovation in manufacturing enterprises or enterprises [59], but there has been no in-depth study on refining heavy-polluting industries and the main choices for carbon reduction. This study enriches the research perspective of green technology innovation to some extent.

Second, this study has opened the “black box” of the process that carbon information disclosure drives green technology innovation of heavily polluting enterprises. This study reveals the intermediate process of carbon information disclosure driving heavily polluting enterprises to carry out green technology innovation and reveals the intermediary role played by financing constraints, which not only provides theoretical support for exploring the effect of carbon information disclosure, but also helps to deepen the theoretical research on corporate financing constraints. Additionally, our research enriches existing research on financing constraints by creating a new indicator system to measure the quality of corporate carbon information disclosure.

### Practical implications

First, our research proves that carbon information disclosure helps improve the level of green technology innovation of enterprises. Therefore, enterprises should have a strategic vision, actively disclose carbon-related information, ensure the transparency of information disclosure, avoid short-term business activities, and disclose high-quality carbon information to promote green technology innovation.

Second, the government and relevant departments should establish and improve a regulatory mechanism for carbon information disclosure, formulate reasonable rules for carbon information disclosure, and urge enterprises to comply with corresponding regulations on carbon information disclosure[60]. Because carbon information disclosure belongs to the category of voluntary disclosure, the willingness of enterprises to disclose carbon information is weak. Government and relevant departments should establish long-term carbon reduction targets and implementation measures, increasing the carriers and forms of carbon information disclosure, and formulating unified forms or standards of carbon information disclosure[61].

Finally, the government should actively promote coordination between the financial sector and environmental regulatory authorities, establish and improve reward and punishment mechanisms, and stimulate and guide the enthusiasm of enterprises to disclose information [62]. Relevant institutions should develop targeted plans to leverage the incentive effect of carbon information disclosure and provide new impetus for green technology innovation.

## Supporting information

S1 File Detailed data on corporate carbon information disclosure, financing constraints, green technology innovation, and control variables. The data were collected from the China Stock Market & Accounting Research Database (CSMAR) and the China Environmental Statistics Database from 2017 to 2021. This dataset supports the analysis of the relationship between carbon disclosure and green technology innovation.
(ZIP)
